# Importance of early recognition and intervention in Ludwig’s angina by emergency medicine physicians to prevent life-threatening complications

**DOI:** 10.1186/s12245-026-01396-7

**Published:** 2026-10-03

**Authors:** Harrison Phillips, Majid Ali Qureshi, Abdul Jalil, Mehtab Khan, Christy Collins, Omar Hafeez-Bore

**Affiliations:** Alexandra Hospital, Worcestershire Acute Hospitals NHS Trust, Redditch, UK

**Keywords:** Emergency medicine, Intensive care, Critical care, Ludwig’s angina, Airway compromise, Le Mierre’s disease, Dental abscess

## Abstract

**Background:**

Ludwig’s angina is a life-threatening cellulitis that can easily progress to airway compromise. Significant infection, often from periodontal abscesses of the molar teeth, may cause superior and posterior displacement of the tongue compromising patient’s airways if not identified and treated promptly. Early recognition and clinical diagnosis, with or without imaging and blood cultures, and antibiotic treatment can help prevent rapid airway compromise, systemic infection and Le Mierre’s disease (a rare life-threatening complication). Given that emergency departments are often the first point of contact, maintaining a high index of suspicion and acting decisively can significantly improve patient outcomes.

**Case Presentation:**

We report a case of a 79-year-old male presenting with a swollen neck progressing over the course of 24 h. This gentleman presented to our emergency department with a superior and inferior swelling to the mandible, a change in voice, and displacement of his tongue. Clinical examination showed a trismus of 1.5 cm, a superiorly and posteriorly displaced tongue, and a tender dental abscess of the lower molars. From examination a clinical diagnosis of Ludwig’s angina was made, later confirmed on CT head and neck. A suspicious incidental finding of an internal jugular embolus on the background of dental infection was also suspicious for an early presentation of Le Mierre’s disease. Intravenous antibiotics were promptly started. Intensive care team was contacted to support the patient’s airway and facilitate transfer to our intensive care unit (ICU). Maxillofacial team was also notified and following antibiotics and tooth/abscess removal the patient made a full recovery and has no repeat presentation since.

**Conclusion:**

This case highlighted and emphasised the importance of early recognition of neck swelling by emergency physicians to prevent progression of airway compromise and Le Mierre`s disease. Assessing emergency doctors must be able to identify, diagnose, treat and escalate Ludwig’s angina secondary to periodontal abscesses prior airway compromise or systemic infection.

## Introduction

Ludwig’s angina is an airway emergency. The condition is a rare but rapidly progressing serious, potentially life threatening, cellulitis of the tissues in the floor of the mouth. Ludwig’s angina presents most commonly following periodontal abscesses especially involving the molar teeth [1]. A route of infection into the sub mylohyoid space is facilitated by the inferior extension of the molar teeth below the mylohyoid line of the mandible. Progression of infection typically takes a superior course from the submaxillary space into the sublingual space. This disease progression ultimately causes induration of the floor of the mouth and can elevate and posteriorly displace the tongue causing acute severe airway compromise [2]. This arises due to pressure-volume dynamics similar to compartment syndromes found elsewhere in the human body. Less common but important causes include oral trauma, infections secondary to oral malignancy, sialadenitis and pericoronitis. Risk factors for Ludwig’s angina may include but are not limited to oral piercings, dental infection, chronic alcohol and injected drug use, diabetes mellitus, poor nutrition and male gender [1]. Mortality may be as high as 50% where treatment is not initiated. Airway oedema has been documented to progress rapidly within the first hour of presentation, owing to importance of early management. Where we see adequate treatment mortality is nearer to 8%, with most fatalities relating to airway compromise [3]. What initially begin as a novel or repetitive dental abscess most commonly, often presents with fever, lethargy, and malaise. Trismus, drooling and dysphagia are often worrying signs, reflecting a more severe infection. Where trismus is present, this often suggests spread of infection into the parapharyngeal space causing increasing discomfort in mouth opening. In advanced cases, patients have been noted to lean forward adopting a tripod position to maximise airway diameter in situations where stridor, amongst other respiratory distress signs, are present [1]. Whilst widely accepted as a clinical diagnosis, CT of the neck with contrast is useful in exhibiting signs such as muscle oedema, increased attenuation of subcutaneous fat, soft tissue gas, airway compromise, and loss of fat planes in submandibular spaces. The use of CT, when combined with clinical examination for deep neck infections, has been shown to be 95% sensitive and 80% specific, and remains the imaging modality of choice [4].

## Case presentation

A 79-year-old male presented to the emergency department in the early hours of the morning with worsening left-sided facial swelling. He had a prior tooth abscess 10 weeks earlier, which resolved following a course of oral antibiotics. The swelling during this presentation developed gradually over the course of a week, with more noticeable progression in the 24 h prior to admission. The patient also reported a three-day history of a hoarse voice and a raised tongue. He was able to speak in full sentences with clear articulation despite the hoarseness and maintained his oxygen saturations independently. His vital signs on presentation demonstrated a blood pressure of 168/89, a heart rate of 85, and a temperature of 36.4 degrees. Otherwise, he felt well and reported no additional symptoms. He had been scheduled for a tooth extraction later that day, but due to increasing pain and swelling, he felt it was more appropriate to attend the emergency department (ED).

### Clinical findings

**Fig. 1:**
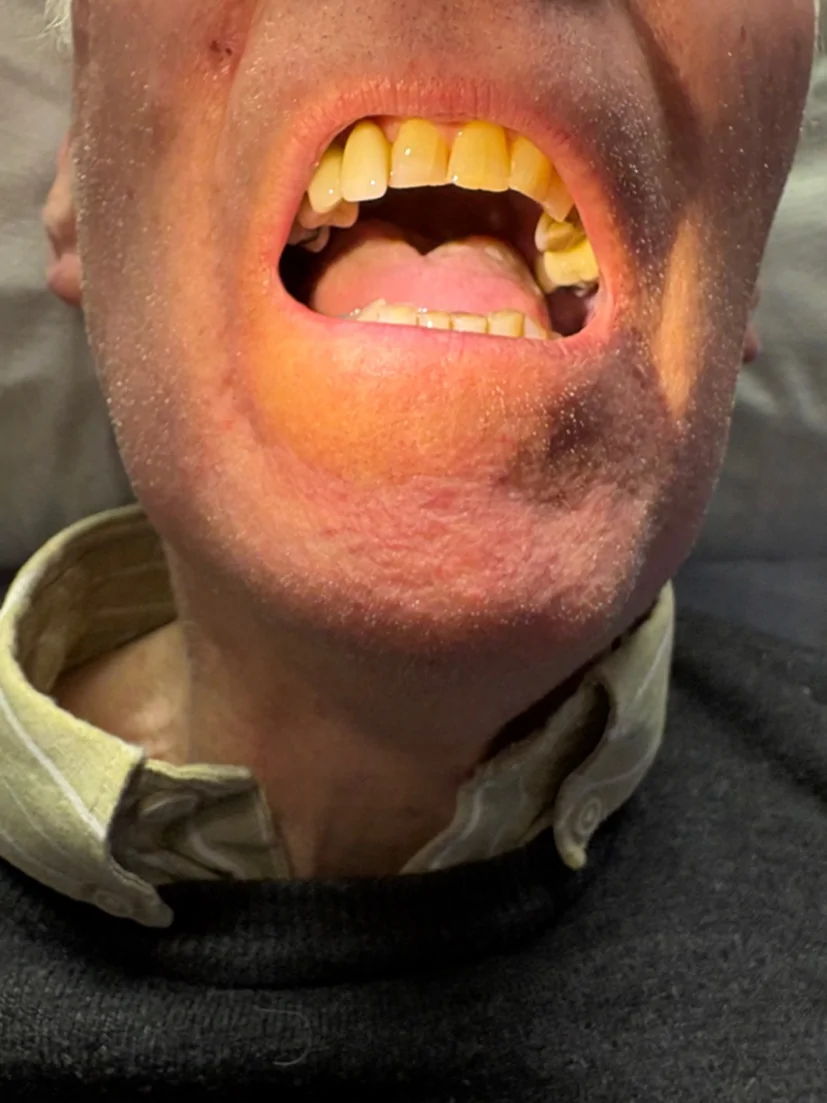
Left neck swelling demonstrating superior elevation of the tongue and blinding of the uvula behind. Marginal spread of infection across the midline of the neck. Photo of our patient taken with consent for use and publication

On clinical examination from the end of the bed, there was noticeable swelling both above and below the level of the mandible. The swelling was firm and tender, with tenderness extending superiorly toward the ear. Palpation of the left lower gum line elicited pain, particularly around the area of swelling near the lower molars. Trismus was present, with a maximal mouth opening of 1.5 cm (Fig. 1). The uvula was just visible behind the tongue, which was mildly displaced superiorly and posteriorly by the underlying infection. There was no evidence of swelling crossing the midline while the patient was in the emergency department. Swallowing was not compromised at this time. There were no signs of external injury, and the remainder of the examination was unremarkable for the patient’s age.

### Diagnostic assessment

**Fig. 2:**
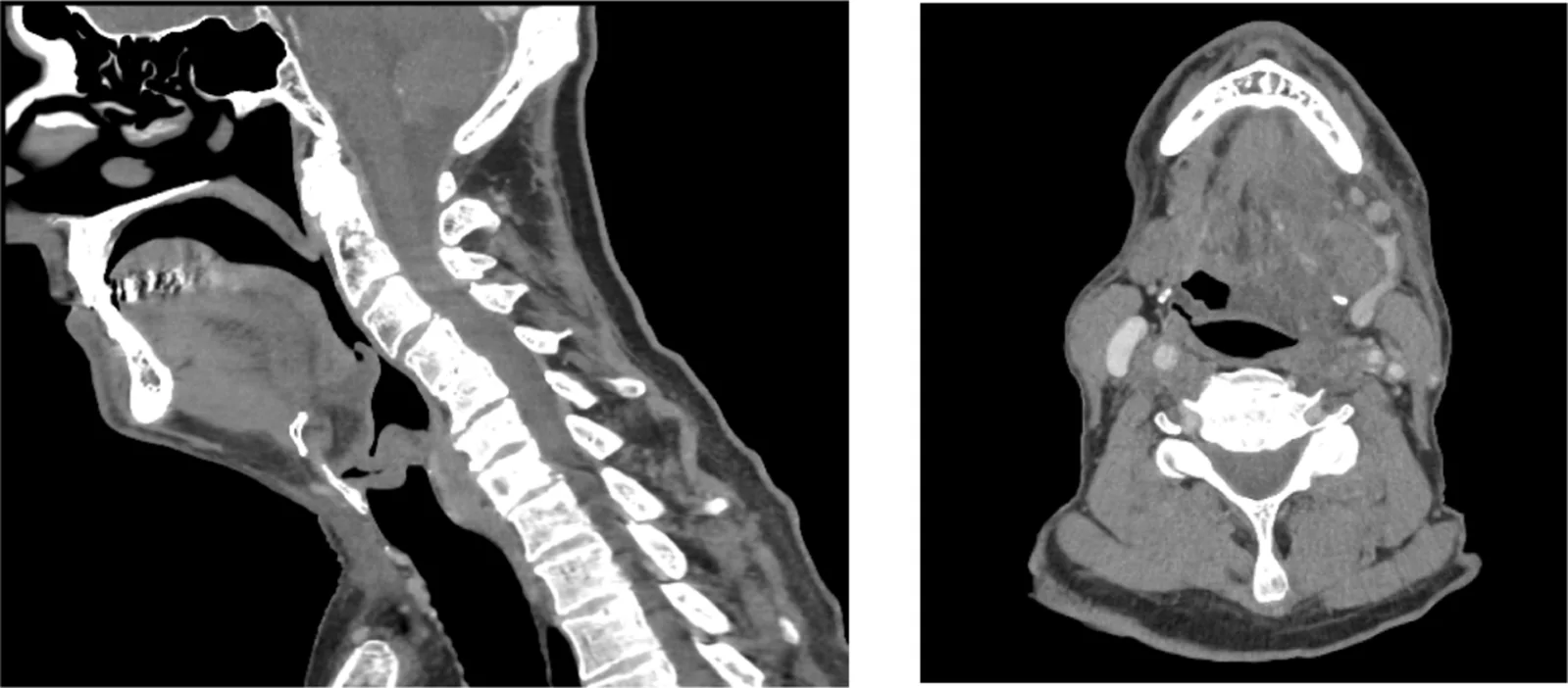
CT findings from this case showing predominantly left-sided multi-spatial diffuse inflammatory process with associated oropharyngeal airway narrowing – features in line with Ludwig’s angina

**Fig. 3:**
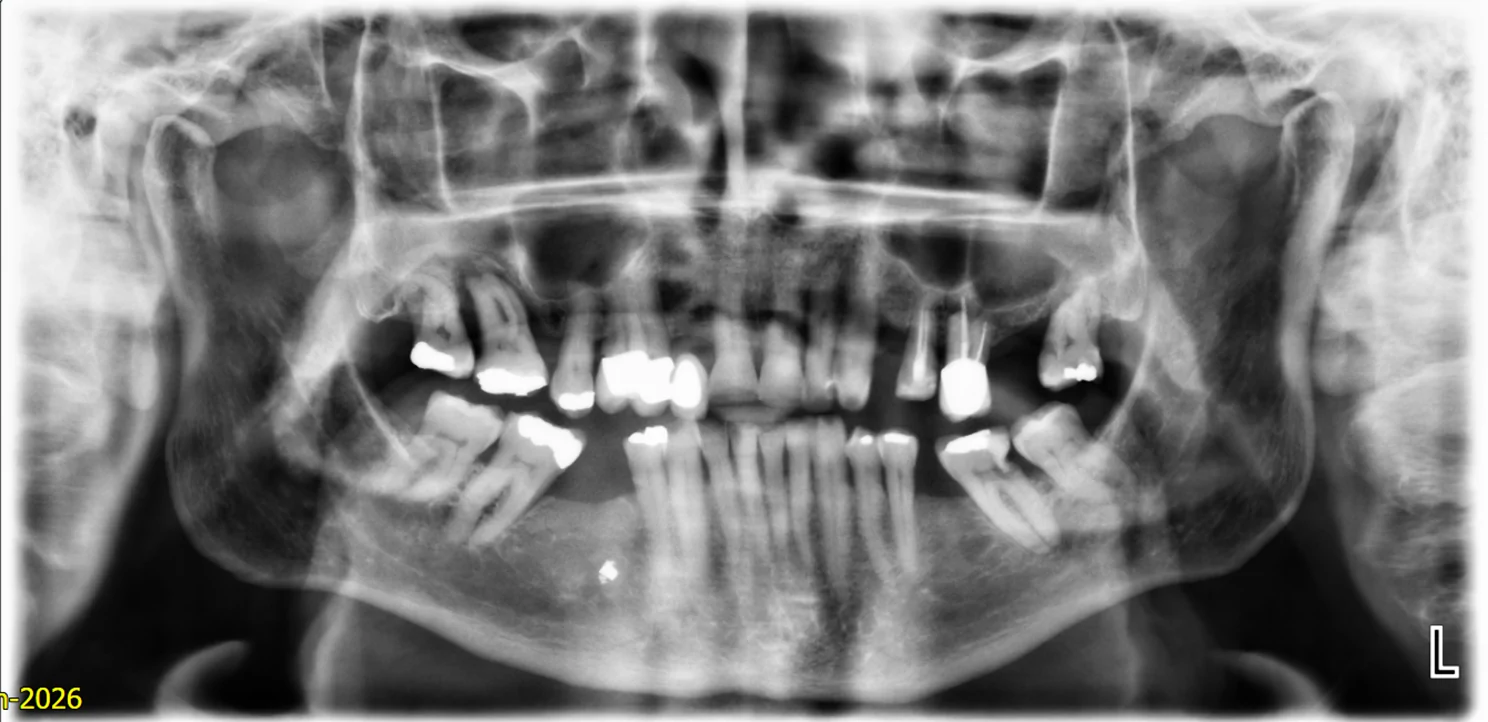
Orthopantomogram used in combination of examination findings and CT, to identify LL7 as the source of infection by ENT / Maxillofacial specialties involved in the case

Although Ludwig’s angina is classically a clinical diagnosis, in this case it was supported by blood tests and imaging due to such an early presentation [4]. Examination, as described above, revealed swelling visible from the end of the bed both above and below the level of the mandible, voice changes suggestive of airway involvement, superior and posterior displacement of the tongue, and a clear source of infection in the lower left molars—findings consistent with the disease’s pathophysiology [5]. Despite pain limiting mouth opening, manipulation of the patients head, use of pen torch, history of recurrent infections in lower left molars, and localisation of pain, a clear source was suspected prior to imaging, later confirmed with help from CT and Orthopantomogram scans (Figs. 2 and 3). A contrast-enhanced CT scan of the neck was performed, which demonstrated a predominantly left-sided, multi-spatial diffuse inflammatory process (mainly involving the submandibular gland), associated with oropharyngeal airway narrowing, in keeping with Ludwig’s angina (Fig. 2). A potential source of infection was identified as a small periapical lucency in the left posterior molar. The diagnosis of Ludwig’s angina in this case was therefore made based on both clinical examination *and* CT findings. Additionally, an incidental left internal jugular vein thrombus was identified, along with concern for intracranial venous thromboembolism involving the left transverse sinus.

### Therapeutic intervention

Due to possible airway compromise, the patient was administered Intravenous penicillin (Co-Amoxiclav 1.2 g), Metronidazole 500 mg and Dexamethasone 6.6 mg on urgent basis as per local guidelines along with Paracetamol for analgesia. Shortly after administration of the intravenous steroid, patient reported an improvement in pain and movement of the mouth. His vital signs shifted to a blood pressure of 134/77, heart rate of 85, and temperature of 36.6 degrees, showing a correction of blood pressure since before treatment begun, but no significant change to the rest, owing to how despite concerning changes on CT, the patients numerical observations remained mostly not concerning. At the earliest convenience the Maxillofacial team was contacted who recommended ENT input for scope and airway assessment. A lack of ENT on site resulted in ITU involvement for airway assessment, before Maxillofacial assessment could be completed. A combination of input guided this patient’s management. Consultant radiologist opinion deemed no discrete collection in the floor of the mouth, with no retropharyngeal abscess or collection. Examination of the head and neck showed good airway patency, slightly oedematous floor of the mouth, but no palpable collection. Mouth opening had eased since initiation of intravenous antibiotics and steroid. It was therefore opted for tooth extraction as an emergency, rather than routine as previously planned in the community referral, without need for specific surgical drainage.

Following assessment, LL7 and LL8 teeth were identified as associated with infection causing airway compromise. Due to clinical improvement, LL7 tooth was able to be extracted under local anaesthetic to remove main source of infection.

### Follow up and outcome

On discharge, oral apixaban was switched to treatment-dose enoxaparin following the incidental finding of an internal jugular vein thrombosis on CT, in accordance with haematology team advice. An outpatient follow-up appointment with haematology was arranged. The patient was also prescribed oral co-amoxiclav (625 mg three times daily for 7 days) by the ITU team. The patient was treated promptly before any serious complications developed and made a significant recovery shortly after admission.

## Discussion

### Literature review

With relevance to this case, previous case reports were reviewed and compared. In most cases of Ludwig’s angina, bacteria from the oral cavity—such as *Streptococcus viridans*, *Staphylococcus aureus*, and *Staphylococcus epidermidis*—have been identified as common causative organisms. The anaerobe *Fusobacterium* is also recognised as an important pathogen, particularly in infections arising from dental abscesses [6]. Although not essential for establishing the diagnosis, with this being widely seen as a clinical diagnosis, blood cultures were not obtained in this patient to confirm the causative organism. Instead, antimicrobial sensitivities were inferred from the patient’s previous response to antibiotics during an earlier dental infection. We recognise the importance that microbiological findings would have had in this case, given severity of the infection and the suspected internal jugular vein thrombosis/Lemierre’s syndrome. As we are used to seeing diffuse neck swelling and systemic upset, this early presentation appeared much tamer, leading to the patient being seen in time order, and not as an emergency. Following thorough examination and blood tests, and due to the reassuring appearance of the patient from the end of the bed, we opted for a CT in this case to help aid diagnosis to ensure it was not a representing simple tooth infection, giving the patients history of recurring dental infection. The radiologist phoned the result through, leading us to immediately call ENT / ITU to ensure the airway was secure as a priority. In the same time the patient was started on IV antibiotics and steroid, and blood cultures were missed from this patient initial workup. This could have been due to reassuring infection markers, availability of recent prior sensitivities, and absence of temperature or signs of systemic upset in addition to ensuring airway prioritisation. This remains a limitation of this case, albeit remaining a widely recognised clinical diagnosis.

As supported by current literature, prompt identification of airway compromise and early treatment of the underlying cause remain crucial in the successful management of Ludwig’s angina, as it is a medical emergency.

This case is particularly notable due to the incidental findings identified on CT imaging of the head and neck. As Ludwig’s angina is widely recognised as a clinical diagnosis, the routine imaging of this anatomy is often not used in the *diagnostic* evaluation of the case. While it can be, and remains, an important tool to evaluate severity and urgency of possible airway compromise, it is often not necessary to diagnose a presentation of Ludwig’s angina. In our case, due to the recurrent infections of the tooth, the patient could have presented with another common tooth infection where the pain demonstrated subpar control in the community. Because of the patient appearing well from the end of the bed, with reassuring bloods and vital signs, it was necessary to use imaging to help *rule out* Ludwig’s angina. It was therefore essential and important in this case especially, whereby in early presentations swelling is not marked on examination, so imaging of the airway provides both diagnostic benefit and helps guide diagnosis *and* management.

This offers insight into how early presentations of this case require an additional awareness and clinical accruement compared to usual presentations, as clinical examination did not reflect severity of CT findings and airway compromise this patient had. This is a potentially very dangerous situation for a patient to find themselves in in the waiting room of an emergency department and therefore warrants attention from emergency physicians.

A non-occlusive thrombus in the left internal jugular vein, likely secondary to dental infection, raised concern for potential progression to Lemierre’s disease. Although not yet a septic thrombus, this finding warranted close attention. Lemierre’s disease, previously referred to as post-anginal septicaemia, is a rare but recognised complication of Ludwig’s angina and is commonly associated with bacteraemia caused by organisms such as *Fusobacterium necrophorum* [7]. Management typically includes intravenous antibiotics, therapeutic anticoagulation, and treatment or removal of the underlying source of infection. As this treatment approach overlaps significantly with that of Ludwig’s angina, the risk of progression to this complication was minimised, contributing to a favourable outcome in this patient.

This case highlights the importance of maintaining a high index of suspicion for potential complications of Ludwig’s angina, particularly in elderly patients and those with comorbidities. Early recognition of clinical deterioration, prompt initiation of intravenous therapy, and timely airway assessment are essential and can lead to full recovery and positive patient outcomes, as demonstrated in this case.

## Conclusion

- Ludwig’s angina remains a clinically diagnosed medical emergency due to the risk of airway compromise. However early presentations can be subtle, and hard to differentiate from other common diagnoses, such as recurrent dental infections such as in our patient.
- Clinical examination and vital signs may not accurately depict impending medical emergencies in patients with less severe presentations.
- All emergency doctors should be familiar with head and neck (including airway) emergencies and be able to recognise Ludwig’s angina clinically *and* early.
- Prompt escalation for airway management, along with early initiation of intravenous antibiotics as per local guidelines, steroids, and appropriate analgesia, is essential when the diagnosis is suspected.
- Emergency doctors at all levels should be able to assess for airway compromise and be confident and competent in escalating care if patient deterioration is suspected.
- Education of emergency staff is necessary to ensure awareness and understanding of potential complications of head and neck infections, such as Lemierre’s syndrome.

## Data Availability

All data generated or analyzed during this study is included in the publication.

## Abbreviations

CT: Computerised tomography
ITU: Intensive treatment unit
IV: Intravenous
LL 7: Left lower 7
LL 8: Left lower 8
ENT: Ear, nose, throat
